# Equal receipt of specialized palliative care in breast and prostate cancer: a register study

**DOI:** 10.1007/s00520-022-07150-y

**Published:** 2022-06-14

**Authors:** Jenny Bergqvist, Christel Hedman, Torbjörn Schultz, Peter Strang

**Affiliations:** 1 Department of Medical Epidemiology and Biostatistics, Karolinska Institutet, Stockholm, Sweden; 2 Breast Center, Department of Surgery, Capio St Gorans Sjukhus, St Görans plan 1, 112 19 Stockholm, Sweden; 3 Department of Molecular Medicine and Surgery, Karolinska Institutet, Stockholm, Sweden; 4 R & D Department, Stockholms Sjukhem Foundation, 102 26, P. O. Box 12230, Stockholm, Sweden; 5 Department of Oncology-Pathology, Karolinska Institutet, Stockholm, Sweden; 6 Regional Cancer Centre Stockholm-Gotland, Stockholm, Sweden

**Keywords:** Breast cancer, Prostate cancer, Metastasis, Quality of life, Registries, Cancer management

## Abstract

**Purpose:**

There are inequalities in cancer treatment. This study aimed to investigate whether receipt of specialized palliative care (SPC) is affected by typical female and male diagnoses (breast and prostate cancer), age, socioeconomic status (SES), comorbidities as measured by the Charlson Comorbidity Index (CCI), or living arrangements (home vs nursing home residence). Furthermore, we wanted to investigate if receipt of SPC affects the place of death, or correlated with emergency department visits, or hospital admissions.

**Methods:**

All breast and prostate cancer patients who died with verified distant metastases during 2015–2019 in the Stockholm Region were included (*n* = 2516). We used univariable and stepwise (forward) logistic multiple regression models.

**Results:**

Lower age, lower CCI score, and higher SES significantly predicted receipt of palliative care 3 months before death (*p* = .007–*p* < .0001). Patients with prostate cancer, a lower CCI score, receiving palliative care services, or living in a nursing home were admitted to a hospital or visited an emergency room less often during their last month of life (*p* = .01 to < .0001). Patients receiving palliative care services had a low likelihood of dying in an acute care hospital (*p* < .001). Those who died in a hospital were younger, had a lower CCI score, and had received less palliative care or nursing home services (*p* = .02– < .0001).

**Conclusion:**

Age, comorbidities, and nursing home residence affected the likelihood of receiving SPC. However, the diagnosis of breast versus prostate cancer did not. Emergency room visits, hospital admissions, and hospital deaths are registered less often for patients with SPC.

## Introduction

Breast and prostate cancer are the two most common cancer diagnoses in Sweden, with approximately 9000 and 10,000 new cases annually, respectively. The 10-year survival rate is 86% for breast cancer and 88% for prostate cancer [1]. The mean survival for patients with disseminated disease is approximately 3 years for both breast [2] and prostate cancer [3].

The last year of life is preceded by up to several years of oncologic treatments, with many patients being able to live a relatively normal life until the last months of life. The disease trajectory is accompanied by several and sometimes complex symptoms and symptom clusters involving pain, nausea, pleural and peritoneal effusions, recurrent infections, dyspnea, cancer fatigue, loss of appetite, and cachexia [4–11].

The symptoms differ between the diagnoses and according to metastatic site [4, 6–8]. Additional metastatic sites (for pleural, peritoneal, lung, and brain metastases, for example) are more common in breast cancer patients, which results in a greater variety of symptoms compared to metastatic prostate cancer, for which fatigue and pain are the most dominant symptoms [4, 6–10]. Palliative care is important for symptom relief and improved quality of life for patients in these large cohorts, and might also prolong survival [12, 13].

In the Swedish health care system, oncologic treatment is offered by hospitals, whereas symptom control, psychosocial support, and other palliative measures are optimally offered by specialized palliative care services (SPC). In the Stockholm Region (the Stockholm County Council), SPC is mainly offered with the aid of ASIH (highly specialized palliative home care teams) that operate 24 h a day, 7 days a week, and are staffed by physicians, registered nurses, and paramedics. ASIH takes care of all medical aspects, with any needed help in daily activities taken care of by the municipality’s home service. It is possible for patients living alone to receive palliative home care and die at home [14].

Patients with chronic medical conditions, including advanced cancer, may be referred for specialized palliation of complex symptoms when general palliative care through primary care or specialist care, such as oncology, is not sufficient. Both patients and their families are very satisfied with the symptom control and support offered by such home care services [15]. In most cases, the home care teams can offer inpatient palliative care at special units outside the acute hospitals when needed for palliation of symptoms or at end of life, but a majority of patients prefer to die at home, provided they receive adequate medical and other support [16, 17].

The aim of ASIH is to take care of all medical aspects at home so that the patients do not have to go to a hospital except for visits to the Oncology Department for chemotherapy, for example.

General palliative care, in contrast to specialized palliative care, can be provided in all health care settings such as hospitals or nursing homes but also at home by district nurses supported by general practitioners during office hours [18].

The distribution of SPC should be equal regardless of diagnosis, age, sex, socioeconomic status (SES), or comorbidity. This is not always the case, and studies have shown differences in distribution related to these factors [19–21].

## Aims

The primary aim was to study if any of diagnosis (breast versus prostate cancer), age at death, sex, SES, comorbidity according to the Charlson Comorbidity Index (CCI), and being resident in a nursing home affects the likelihood probability of receiving SPC. Secondary aims were to study if receiving SPC reduces hospital deaths, and if it correlates with emergency visits, or hospital admissions.

## Patients and methods

The methods and results are reported, whenever possible, based on the Strengthening the Reporting of Observational Studies in Epidemiology (STROBE) criteria [22].

### Study design

This descriptive registry study is based on data from VAL, the Stockholm Region’s central data warehouse with registers for outpatient visits to hospitals and hospital admissions. Data from a 5-year period (2015–2019) were retrieved and various aspects of healthcare consumption were compared among those who died from either metastatic breast or prostate cancer. For each patient included in the analysis, data on palliative care services received were collected for the 3 months preceding the date of death. Registrations of emergency room visits and hospital admissions were from the last month of life. Data were further analyzed according to age, sex, living arrangements (resident in a nursing home versus all others), and SES using Mosaic [23–25]. Stockholm County is divided into approximately 1300 areas that are classified according to the Mosaic system. Mosaic provides socioeconomic status data and allows the Stockholm Region to define and allocate different areas of residence within the County of Stockholm to one of three different socioeconomic classes, namely, Mosaic group1, group 2, and group 3 where 1 = high SES and 3 = low SES. The designations are mainly based on income and education for the population in that area, but also factors in more than 40 other elements, such as cultural aspects, lifestyle, and living arrangements.

### Study population

All patients over the age of 18 who died during the years 2015 to 2019 with a main diagnosis of breast cancer (ICD-10 code C50) or prostate cancer (C61) were included, provided they also had a secondary diagnosis of metastatic disease (C78–C79).

### Variables

Outcome measures were the receipt of SPC, emergency room visits, admission to acute care hospitals, and acute care hospitals as place of death. Explanatory variables were age at death, sex, living arrangement (nursing home versus all others), CCI as a measure of comorbidity [26], and Mosaic group as a measure of area-based SES.

### Selection bias

#### Dropouts

As reporting data to VAL is an obligation for each clinic/care unit in the region and a basis for economic compensation, the data are close to complete, with few missing values.

#### Nursing home residents

Nursing homes are run by municipalities, but physicians are employed by the Region (county council). Therefore, residents were identified through registrations of medical interventions by physicians, as such care is exclusive to nursing home residents and has a unique, identifiable code.

### Study size

This study covered all cohorts, i.e., all deaths (all causes) during the years 2015–2019. Therefore, no power calculations were performed.

### Statistical methods, missing data

T-tests and chi-squared tests were used to compare the proportions. The few missing data points were not substituted. Initially, univariable logistic regression analyses were performed, followed by stepwise (forward) multiple regression. Stepwise regression was chosen as all the studied variables were considered relevant. The SAS version 9.4 was used for statistical analysis.

### Ethics

The study was approved by the Regional Ethics committee (EPN, 2017/1141–31/4).

## Results

During the five consecutive years 2015–2019, a total of 1062 women with a breast cancer diagnosis and 2161 men with a prostate cancer diagnosis died. The mean ages for the two groups were 71.3 years and 80.3 years, respectively (*p* < 0.0001). As prostate cancer sometimes is a concomitant diagnosis in an elderly person who dies from other causes, the subsequent analyses were delimited to those patients who died with known distant metastases, which resulted in 950 women with metastatic breast cancer and 1566 men with metastatic prostate cancer. The mean ages for the metastatic breast cancer and prostate cancer groups were 69.7 and 78.7 years, respectively (*p* < 0.0001). See Table 1*.*

**Table 1:** Characteristics and care utilization for 2516 patients who died in breast cancer (*n* = 950) or in prostate cancer (*n* = 1566)

Characteristics and care utilization	Total	Breast	Prostate	*p*-value
**Deaths**	2516	950	1566	**-**
**Age** (SD)**,** years	75 (12)	69.7 (13.9)	78.7 (8.8)	< .0001
**Age groups**				
18–64 years, *n* (%)	381 (15)	298 (31)	83 (5)	< .0001
65–74 years, *n* (%)	727 (29)	292 (31)	435 (28)	0.113
75–84 years, *n* (%)	813 (32)	222 (23)	591 (38)	< .0001
85 years or older, *n* (%)	595 (24)	138 (15)	457 (29)	< .0001
**Access to SPC**, *n* (%)	1971 (78)	783 (82)	1188 (76)	< .0001
**Care in nursing homes**, *n* (%)	318 (13)	100 (11)	218 (14)	0.013
Age, nursing home residents (years, SD)	83 (8)	81.4 (10)	84.4 (7)	0.005
**Charlson Comorbidity Index (CCI)**				
0–1, *n* (%)	1765 (70)	761 (80)	1004 (64)	< .0001
≥ 2, *n* (%)	751 (30)	189 (20)	562 (36)	< .0001

*T*-test was used for comparison of age. Chi-2 test was used for comparison of proportions

*SD* standard deviation

*SPC* specialized palliative care

### Receipt of specialized palliative care

#### Univariable analyses

Among 2516 patients who could be evaluated, 78% had received SPC at some point during their last 3 months of life, 82% for breast, and 76% for prostate cancer (*p* < 0.0001). See Table 1. In a univariable analysis, patients with metastatic breast cancer were more likely to receive SPC, with an odds ratio (OR) of 1.49 (1.22–1.83), *p* < 0.0001. See Table 2. When studying both groups jointly, a younger age, living in a high SES area (Mosaic group 1), and having a lower CCI score were additionally associated with higher ORs in univariable comparisons (*p* = 0.008 to *p* < 0.0001 in most comparisons). See Table 2.

**Table 2:** Received specialized palliative care. Variables related to receipt of palliative care services. Odds ratios (OR) for different variables, based on *n* = 2516 observations. The multivariable analysis was performed as a stepwise multiple logistic regression. Diagnosis lost its statistical significance when other variables were entered into the model

Variable	Univariable analysis		Multivariable analysis	
	OR (95% CI)	*p*-value	OR (95% CI)	*p*-value
Diagnosis				
Breast cancer*.^1^	1.49 (1.22–1.83)	< .0001		
Prostate cancer*.^1^	Ref			
Socioeconomic status				
Mosaic group 1	1.40 (1.10–1.79)	.008	1.42 (1.10–1.83)	.007
Mosaic group 2	1.20 (0.96–1.50)	.10 (ns)	1.15 (0.92–1.45)	.22
Mosaic group 3	Ref		Ref	
Age groups				
18–64 years	3.84 (2.72–5.43)	< .0001	3.31 (2.32–4.71)	< .0001
65–74 years	3.11 (2.39–4.04)	< .0001	2.92 (2.24–3.82)	< .0001
75–84 years	2.02 (1.59–2.56)	< .0001	1.97 (1.55–2.51)	< .0001
85 years or older	Ref		Ref	
CCI				
0–1	1.99 (1.64–2.43)	< 0.0001	1.64 (1.34–2.02)	< .0001
≥ 2	Ref		Ref	
Nursing home resident				
Yes	0.15 (0.12–0.19)	< 0.0001	0.19 (0.15–0.25)	< .0001
No	Ref		Ref	

^*1^Only patients with distant metastases were included

*CCI* Charlson Comorbidity Index

*OR* odds ratio

*CI* confidence interval

#### Multivariable models

In a final stepwise logistic regression model that included diagnosis, age groups, SES, CCI, and residence in a nursing home, all variables except for diagnosis retained their predictive value with almost similar values as in the univariable analyses. See Table 2.

In a separate stepwise multiple logistic regression model, in which the 318 who were residents in nursing homes were excluded, the same variables were still significant, but the odds ratios for the age groups were lower, with OR values between 1.47 and 1.90 (data not shown in tables).

### Emergency room visits during the last month of life

In total, 40% had at least one emergency room visit during their last month of life (39% for breast and 41% for prostate cancer patients), indicating that about 60% had no need for acute emergency room visits even during their last month of life. Among patients who received SPC, 36% had at least one emergency room visit compared with 55% among those without SPC (< 0.0001). See Table 3.

**Table 3:** Emergency room visits (last month), hospital admissions (last month), and hospital as place of death, with and without access to specialized palliative care

Care utilization	Total	With access to SPC	Without access to SPC	*p*-value^*1^
**Emergency room visits**				
Breast cancer, *n* (%)	368/950 (39)	290/783 (37)	78/167 (47)	.0199
Prostate cancer, *n* (%)	645/1566 (41)	422/1188 (36)	223/378 (59)	< 0.001
All patients, *n* (%)	1013/2516 (40)	712/1971 (36)	301/545 (55)	< 0.001
**Hospital admissions**				
Breast cancer, *n* (%)	464/950 (49)	367/783 (47)	97/167 (58)	.0085
Prostate cancer, *n* (%)	668/1566 (43)	441/1188 (37)	227/378 (60)	< 0.001
All patients, *n* (%)	1132/2516 (45)	808/1971 (41)	324/545 (60)	< 0.001
Hospital as place of death^*2^				
Breast cancer, *n* (%)	144/950 (15)	62/783 (8)	82/167 (49)	< 0.001
Prostate cancer, *n* (%)	245/1566 (16)	92/1188 (8)	153/378 (41)	< 0.001
All patients, *n* (%)	389/2516 (15)	154/1971 (8)	235/545 (43)	< 0.001

^*1^*p*-value between those with and without specialized palliative care. Comparisons are done with chi-2 tests

^*2^Hospital as place of death does not include geriatric wards. Three percent of all patients died in a geriatric ward

In univariable analyses, receipt of SPC strongly reduced emergency room visits (OR 0.46 (0.38–0.56), *p* < 0.0001). Other variables associated with a reduction in such visits included living in a socioeconomically affluent area (Mosaic group 1), being younger, and having lower CCI values. See Table 4.

**Table 4:** Emergency room visits. Variables that predicted the need for emergency room visits during the patients’ last month of life. Odds ratios (OR) for different variables, based on *n* = 1013/2516 observations. In the stepwise multiple logistic regression model, the variables “palliative care,” “nursing home resident,” CCI, and Mosaic groups were entered first. Diagnosis and age groups became non-significant and did not enter the final model

Variable	Univariable analysis		Multivariable analysis	
	OR (95% CI)	*p*-value	OR (95% CI)	*p*-value
Diagnosis				
Breast cancer*.^1^	0.90 (0.77–1.06)	.22 (ns)		
Prostate cancer*.^1^	Ref			
Socioeconomic status				
Mosaic group 1	0.72 (0.58–0.88)	.002	0.76 (0.62–0.94)	.01
Mosaic group 2	0.91 (0.75–1.01)	.32 (ns)	0.97 (0.80–1.17)	.73
Mosaic group 3	Ref		Ref	
Age groups				
18–64 years	0.72 (0.55–0.94)	.02		
65–74 years	0.80 (0.64–0.996)	.04		
75–84 years	0.94 (0.76–1.16)	.55 (ns)		
85 years or older	Ref			
CCI				
0–1	0.69 (0.50–0.95)	< 0.02	0.73 (0.61–0.87)	.0006
≥ 2	Ref		Ref	
Access to palliative care				
Yes	0.46 (0.38–0.56)	< .0001	0.41 (0.33–0.50)	< .0001
No	Ref			
Nursing home resident				
Yes	0.83 (0.65–1.06)	.14 (ns)	0.54 (0.41–0.70)	< .0001
No	Ref		Ref	

^*1^Only patients with distant metastases

*CCI* Charlson Comorbidity Index

*OR* odds ratio

*CI* confidence interval

In a multivariable stepwise logistic regression, diagnosis and age were non-significant, whereas the other variables remained significant. Receipt of palliative care and, especially, being a nursing home resident showed increased significance. See Table 4.

### Admissions to acute hospitals during the last month of life

In total, 45% were admitted to an acute hospital at least once during the last month of life (49% for breast cancer and 43% for prostate cancer). Of those who received palliative care, 41% had at least one admission to an acute care hospital compared with 60% among those without palliative care (< 0.0001). See Table 3.

In univariable analyses, receipt of palliative care reduced the admissions to acute hospitals (OR 0.47 (0.39–0.58), *p* < 0.0001), a result that was also seen for those living in nursing homes, having prostate cancer, living in a high SES area, being older, and having lower CCI values. See Table 5.

**Table 5:** Hospital admissions. Variables that predicted the need for admissions to acute hospitals during the patients’ last month of life. Odds ratios (OR) for different variables, based on *n* = 1132/2516 observations. In the stepwise multiple logistic regression model, the variables “palliative care,” “nursing home resident,” CCI, and Mosaic groups were entered first. Socioeconomic status in the form of Mosaic groups became non-significant and did not enter the final model

Variable	Univariable analysis		Multivariable analysis	
	OR (95% CI)	*p*-value	OR (95% CI)	*p*-value
Diagnosis				
Breast cancer*.^1^	1.28 (1.09–1.51)	.002	1.31 (1.10–1.57)	.003
Prostate cancer*.^1^	Ref		Ref	
Socioeconomic status				
Mosaic group 1	0.82 (0.67–0.999)	.049		
Mosaic group 2	0.88 (0.73–1.06)	.19 (ns)		
Mosaic group 3	Ref			
Age groups				
18–64 years	1.53 (1.18–1.98)	.001	1.50 (1.12–2.02)	.007
65–74 years	1.24 (1.00–1.55)	.05	1.32 (1.04–1.67)	.02
75–84 years	1.24 (0.999–1.53)	.05	1.31 (1.04–1.64)	.02
85 years or older	Ref		Ref	
CCI				
0–1	0.80 (0.67–0.95)	.01	0.76 (0.63–0.91)	.003
≥ 2	Ref		Ref	
Access to palliative care				
Yes	0.47 (0.39–0.58)	< .0001	0.35 (0.28–0.44)	< .0001
No	Ref			
Nursing home resident				
Yes	0.63 (0.49–0.80)	.0002	0.43 (0.32–0.57)	< .0001
No	Ref		Ref	

^*1^Only patients with distant metastases

*CCI* Charlson Comorbidity Index

*OR* odds ratio

*CI* confidence interval

A stepwise multivariable regression model showed that the effect of receiving palliative care and living in a nursing home increased. The effect of having prostate cancer, being younger, and having a lower CCI values remained significant regarding hospital admissions. See Table 5.

### Hospital deaths

In total, 15% died in acute hospitals and 3% died in geriatric wards. Of those 1971 patients who received palliative care, mainly in the form of palliative home care, 8% died in an acute care hospital, whereas the corresponding figure was 43% for those who were never enrolled in palliative care (*p* < 0.0001). See Table 3.

Consequently, a univariable logistic regression revealed that death in a hospital occurred less often for people who received palliative care (OR 0.11 (0.09–0.14), *p* < 0.0001), and also for those living in high SES areas (Mosaic groups 1 and 2), those with lower CCI values, and those residing in nursing homes. See Table 6.

**Table 6:** Acute hospital deaths. Variables that correlated with the 389 (of 2516) deaths in acute hospitals. Odds ratios (OR) for different variables. In the stepwise multiple logistic regression model, the variables palliative care, being nursing home residents, and age groups were entered first. Diagnosis and socioeconomic status (Mosaic groups) became non-significant and were not entered in the final model

Variable	Univariable analysis		Multivariable analysis	
	OR (95% CI)	*p*-value	OR (95% CI)	*p*-value
Diagnosis				
Breast cancer*.^1^	0.96 (0.77–1.21)	.74 (ns)		
Prostate cancer*.^1^	Ref			
Socioeconomic status				
Mosaic group 1	0.65 (0.49–0.86)	.002		
Mosaic group 2	0.70 (0.54–0.90)	.005		
Mosaic group 3	Ref			
Age groups				
18–64 years	1.49 (1.04–2.12)	.03	2.88 (1.87–4.43)	< .0001
65–74 years	1.24 (0.91–1.69)	.18	2.09 (1.44–3.03)	.0001
75–84 years	1.28 (0.94–1.74)	.11	1.81 (1.28–2.58)	.0009
85 years or older	Ref			
CCI				
0–1	0.66 (0.53–0.83)		0.73 (0.56–0.95)	.02
≥ 2	Ref	.0003	Ref	
Access to palliative care				
Yes	0.11 (0.09–0.14)	< .0001	0.07 (0.05–0.09)	< .0001
No	Ref		Ref	
Nursing home resident				
Yes	0.69 (0.48–0.99)	.04	0.22 (0.16–0.36)	< .0001
No	Ref		Ref	

^*1^Only patients with distant metastases

*CCI* Charlson Comorbidity Index

*OR* odds ratio

*CI* Confidence interval

In a stepwise multivariable logistic regression, the impact of receiving palliative care or being resident in a nursing home was strengthened, with fewer hospital deaths. In addition, the impact of age was more pronounced in the multivariable analysis. See Table 6.

## Discussion

Our study shows equal receipt of SPC for the typical female and male cancer diagnoses of breast and prostate cancer, when controlled for age and other relevant variables. However, our results showed inequality with regard to age, comorbidities, and socioeconomic factors. Younger patients, those with lower CCI scores, and persons from more affluent socioeconomic areas (Mosaic group 1) received SPC more often. Emergency room visits, hospital admissions, and acute care hospitals as a place of death were less likely for patients who received palliative care or resided in a nursing home. Utilization of acute hospital services during the last month of life was also partly affected by age, comorbidities, and whether you lived in an area with high or low SES. The reason for these differences may be that SPC leads to fewer symptoms and a reduced utilization of hospital services. However, it might also in total or in part depend on the patient’s choice to receive care at home and/or not wanting to go to a hospital.

In Sweden, healthcare is mainly financed by taxes. Consequently, we expect the distribution of SPC to be based on actual needs, not on age, sex, SES, or comorbidities. Nonetheless, our data show that younger patients as well as patients from more affluent SES areas were more likely to receive SPC, well in line with other cancer studies [19, 20, 27, 28]. However, we do not know whether patients living in areas with high SES are referred to SPC to a greater extent or more often accept admission to SPC in comparison with patients from areas with low SES. Furthermore, we have analyzed the registration of SPC being received, but we do not have access to all possible patient-related factors that might further explain why some people did not receive SPC.

In our study, female patients (breast cancer) and male patients (prostate cancer) had similar odds ratios of receiving SPC when controlled for age, which differs from a large Danish study encompassing all cancer diagnoses. In that study, women were more likely to receive SPC [19].

In end-of-life situations, unnecessary, burdensome acute visits to emergency rooms should be avoided [29]. We found that receiving SPC significantly reduced the odds ratios of emergency room visits, well in line with other studies [29]. In a meta-analysis with more than 1 million patients from 5 countries, the odds ratio was 0.43 for emergency room visits during SPC [30], a result that is well in line with the value of 0.41 in our study. In the meta-analysis, a higher CCI score and a lower SES were also associated with more emergency room visits, also in line with our findings. As expected, nursing home residents had fewer emergency room visits in our study, when controlling for diagnosis, SES, age, and comorbidities, a result that is also corroborated in similar studies [29].

Multiple hospital admissions, as well as acute care hospitals as a place of death, are associated with more aggressive treatments and poorer quality of life, as most patients would prefer to die at home [16, 17]. As expected, receiving palliative care was by far the strongest predictor of not dying in an acute care hospital, with a very low OR of 0.07 (0.05–0.09), followed by being a nursing home resident. In a meta-analysis comprising 112 studies, lower SES was associated with hospital deaths [28], a finding that was not confirmed in our data. A possible explanation might be that in Sweden the distribution of SPC is tax-financed and, therefore, many cancer patients die while receiving such services. When controlling for other variables, both higher CCI scores and younger age were associated with hospital deaths.

In total, 78% of the patients in our study received SPC, which is desirable as such services are successful in regard to symptom control and other kinds of support [15]. Moreover, most cancer patients prefer home care and dying at home, when possible, provided high-quality care is offered [16].

Comparisons of hospital deaths and receipt of palliative care are difficult to make across countries because of different settings. One study from Canada showed that 73% of patients dying from cancer had received palliative care from a specialist [31]. These numbers are somewhat lower than ours. Place of death was included in a study from the USA, with 25% of all cancer patients dying in a hospital [32].

As pointed out in a review, palliative home care is not an optimal solution for certain patients and for different reasons [33]. Therefore, SPC services should be offered both at home and in inpatient settings, as is the case in Region Stockholm. One group of patients that receives palliative care less often is those living in nursing homes. Previous studies have shown that patients with cancer living in nursing homes report a high prevalence of pain, reduced symptom treatment [34], and, for those with comorbid dementia, a high prevalence of neuropsychiatric symptoms [35]. This emphasizes the need for the distribution of palliative care also in nursing homes, to enable adequate symptom management.

### Strengths and limitations

As reporting to the VAL databases is mandatory, the data have very few missing values.

A limitation of this study is that the diagnosis for each patient was not based on the death certificate, but on the primary diagnosis during the last episode of care. However, the probability that breast or prostate cancer was the main diagnosis is strengthened by our selecting participants with concomitant occurrence of a diagnosis of secondary tumors (metastases). Therefore, patients with indolent tumors were excluded from the final analysis.

We used Mosaic for the SES variable, which is area-based and not based on individual factors. Furthermore, we have analyzed received SPC in our registry study, but we did not have access to all patient-related factors that may explain why or why not a patient received SPC.

## Conclusions

Younger patients living in high SES areas with lower CCI scores are more likely to receive specialized palliative care, whereas people residing in nursing homes seldom do, despite disseminated cancer disease. Those who do receive palliative care have fewer hospital admissions and visits to the emergency room, and they seldom die in acute care hospitals. In order to improve the quality of life for all patients with metastatic breast and prostate cancer, we need to ensure equal receipt of specialized palliative care. Although patients in nursing homes have troublesome symptoms, increased delivery of palliative care in nursing homes would be recommended by, for example, continuing education that is tailored to the situation in which a resident may have both a cancer and a dementia diagnosis, or by external palliative care teams with special competence.

## Data Availability

All raw data can be available upon request.

## Code availability

NA.
